# Risk factors for recurrent emergency department visits for hyperglycemia in patients with diabetes mellitus

**DOI:** 10.1186/s12245-017-0150-y

**Published:** 2017-07-12

**Authors:** Justin W. Yan, Katherine M. Gushulak, Melanie P. Columbus, Kristine van Aarsen, Alexandra L. Hamelin, George A. Wells, Ian G. Stiell

**Affiliations:** 1 The Division of Emergency Medicine, Department of Medicine, London Health Sciences Centre, London, ON Canada; 2 Schulich School of Medicine and Dentistry, Western University, London, ON Canada; 3 The Department of Emergency Medicine, The University of Ottawa, Ottawa, ON Canada; 4 The Ottawa Hospital Research Institute, Ottawa, ON Canada

**Keywords:** Diabetes mellitus, Emergency medicine, Risk factors, Recurrent visits, Diabetic ketoacidosis, Hyperosmolar hyperglycemic state

## Abstract

**Background:**

Patients with poorly controlled diabetes mellitus may present repeatedly to the emergency department (ED) for management and treatment of hyperglycemic episodes, including diabetic ketoacidosis and hyperosmolar hyperglycemic state. The objective of this study was to identify risk factors that predict unplanned recurrent ED visits for hyperglycemia in patients with diabetes within 30 days of initial presentation.

**Methods:**

We conducted a 1-year health records review of patients ≥18 years presenting to one of four tertiary care EDs with a discharge diagnosis of hyperglycemia, diabetic ketoacidosis, or hyperosmolar hyperglycemic state. Trained research personnel collected data on patient characteristics and determined if patients had an unplanned recurrent ED visit for hyperglycemia within 30 days of their initial presentation. Multivariate logistic regression models using generalized estimating equations to account for patients with multiple visits determined predictor variables independently associated with recurrent ED visits for hyperglycemia within 30 days.

**Results:**

There were 833 ED visits for hyperglycemia in the 1-year period. 54.6% were male and mean (SD) age was 48.8 (19.5). Of all visitors, 156 (18.7%) had a recurrent ED visit for hyperglycemia within 30 days. Factors independently associated with recurrent hyperglycemia visits included a previous hyperglycemia visit in the past month (odds ratio [OR] 3.5, 95% confidence interval [CI] 2.1–5.8), age <25 years (OR 2.6, 95% CI 1.5–4.7), glucose >20 mmol/L (OR 2.2, 95% CI 1.3–3.7), having a family physician (OR 2.2, 95% CI 1.0–4.6), and being on insulin (OR 1.9, 95% CI 1.1–3.1). Having a systolic blood pressure between 90–150 mmHg (OR 0.53, 95% CI 0.30–0.93) and heart rate >110 bpm (OR 0.41, 95% CI 0.23–0.72) were protective factors independently associated with not having a recurrent hyperglycemia visit.

**Conclusions:**

This unique ED-based study reports five risk factors and two protective factors associated with recurrent ED visits for hyperglycemia within 30 days in patients with diabetes. These risk factors should be considered by clinicians when making management, prognostic, and disposition decisions for diabetic patients who present with hyperglycemia.

**Electronic supplementary material:**

The online version of this article (doi:10.1186/s12245-017-0150-y) contains supplementary material, which is available to authorized users.

## Background

Diabetes mellitus is a chronic disease where treatment is directed toward limiting its potentially severe short- and long-term complications. With the increasing prevalence of diabetes in the general population, it is currently the most prevalent chronic disease among all visitors to the emergency department (ED) [1]. In the USA in 2007, the direct medical costs of managing diabetes were US$116 billion and it has been shown that ED utilization rate by people with diabetes is twice of those without diabetes [2, 3]. Furthermore, diabetic patients without access to a primary care physician or who come from a lower socioeconomic background may have higher ED utilization for diabetes management [4–6]. Emergency physicians play a crucial role in managing patients with diabetes, particularly when they present to the ED with acute and potentially severe complications of their disease process [7, 8].

Hyperglycemic emergencies, including diabetic ketoacidosis and hyperosmolar hyperglycemic state, are known to recur in patients with poorly controlled diabetes [7, 9]. While previous studies have examined the factors that predict short-term unplanned recurrent ED visits for all medical conditions [10–13], literature on predictors of recurrent visits specifically for hyperglycemia is considerably more limited. Some studies have attempted to identify risk factors for readmission to hospital or intensive care for severe diabetic ketoacidosis [9, 14], but these were not ED-based studies and the findings may not be generalizable to the outpatient or emergency population. The ability to identify hyperglycemic patients at higher risk of ED recidivism may be useful for clinicians in order to guide management and disposition decisions including closer follow-up and tighter glycemic control.

The primary objective of this study was to identify the predictors of unplanned recurrent ED visits for hyperglycemia in patients with diabetes mellitus within 30 days of an initial hyperglycemic presentation. Secondary outcomes were to describe the frequencies of specialist consultations in the ED, discharge, and hospital or intensive care unit (ICU) admission for hyperglycemia.

## Methods

### Study design and setting

We conducted a health records review of patients presenting to one of four tertiary care EDs (two academic centers each with two geographically distinct sites), with an approximate combined annual census of 300,000 patients. We studied patients with a discharge diagnosis of hyperglycemia, diabetic ketoacidosis, or hyperosmolar hyperglycemic state over a 1-year period (January–December 2014). The study protocol was approved by the Health Sciences Research Ethics Boards at The Ottawa Hospital in Ottawa, Ontario, Canada, and Western University in London, Ontario, Canada.

### Study population

All index visits of adult (≥18 years) ED patients with a final diagnosis of hyperglycemia, diabetic ketoacidosis, or hyperosmolar hyperglycemic state and its related codes under the International Statistical Classification of Diseases and Related Health Problems, 10th Revision (ICD-10), according to the treating physician were eligible to be included in the study. This included patients with either previously known or unknown diabetes and—if known to be diabetic—both type 1 and type 2 diabetes, regardless of whether or not they were insulin-dependent. Patients with co-morbid final diagnoses in addition to hyperglycemia such as infection, cardiac ischemia, or adverse drug reaction were also included. Patients were excluded if their ICD-10 code was incorrect (i.e., the reason for visit was unrelated to hyperglycemia) or if they were initially assessed at a peripheral or community hospital and transferred to the study sites for ongoing management.

### Outcome measures

The primary outcome was the occurrence of an unplanned return ED visit for hyperglycemia within 30 days of the initial index hyperglycemia visit. Secondary outcomes included the frequency of specialist consultations in the ED (e.g. internal medicine, endocrinology, etc.) and describing the patients’ disposition such as discharge from the ED, or admission to the ward or ICU for further inpatient management of hyperglycemia.

### Data collection and analysis

A list of potential patient visits was generated according to ICD-10 diagnostic codes and charts were reviewed for eligibility. Trained research personnel collected data from paper and electronic medical records using a standardized data collection tool (Additional file 1). Electronic records were reviewed to determine if the patient had an unplanned recurrent ED visit for hyperglycemia within 30 days of the index hyperglycemia visit. Details surrounding both visits, including reason for the visit, pertinent clinical findings, results of investigations, physician management, patient disposition, and final diagnoses were collected. Data from the collection tool were then entered into a study-specific Microsoft Excel database (Microsoft Corporation, Redmond, WA). Patient characteristics were summarized using descriptive statistics and 95% confidence intervals using standard equations when indicated. Differences between patient groups were assessed using chi-squared and Student’s *t* test where appropriate. Data elements were chosen with the intent of evaluating variables for model inclusion based on what is known about the epidemiology of the disease process as well as hypothesized relationships between potential independent variables and recurrent hyperglycemia visits. We explored a number of cutpoints for age, vital signs, and most laboratory values.

Univariate analysis of all potential patient risk factors was completed, and clinically relevant variables with a *p* value of 0.10 or less in the univariate analysis were considered for the multivariate models. Multivariate logistic regression models were used to determine predictor variables independently associated with recurrent ED visit for hyperglycemia. Likelihood ratio tests determined appropriate inclusion of variables in the multivariate logistic regression model. Generalized estimating equations (GEE) were used in order to account for unique patients who had multiple visits during the study period. GEE methods are used to develop regression models for correlated data that arise from repeated measures of the same individuals over time [15–20]. After fitting the GEE model, the predicted probabilities were outputted to a separate file and then used to generate a receiver operating characteristic (ROC) curve in “proc logistic” using the outcome variable and the predicted probabilities variable. The area under the ROC curve for the model was determined; although to obtain a 95% confidence interval of the area under the curve, clustering in the data was disregarded. All analyses were performed using SAS 9.3 Software (SAS Institute Inc, Cary, NC, USA).

## Results

**Fig. 1:**
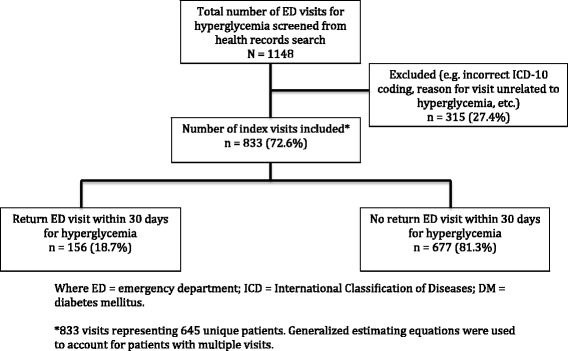
Flow diagram of eligible and included recurrent emergency department hyperglycemia visits

From January–December 2014, a total of 1148 ED visits were screened for eligibility. After applying the exclusion criteria and eliminating those visits that were coded incorrectly, a total of 645 unique patients with 833 total ED visits were ultimately included. Of these visits, 156 (18.7%) were unplanned recurrent ED visits for hyperglycemia within 30 days while 677 (81.3%) did not have a recurrent visit within 30 days (Fig. 1). Characteristics of all hyperglycemia visits, including patient demographics, vital signs, past medical history, and diabetic medications are summarized in Table 1. Table 2 outlines the most common chief complaints for the 833 ED visits for hyperglycemia, the most common being “high blood sugar”, followed by nonspecific complaints of being “dizzy, weak, or unwell” and “nausea and/or vomiting.”

**Table 1:** Characteristics of included hyperglycemia visits organized by whether they had recurrent visits to the ED within 30 days for hyperglycemia (*n* = 156) or not (*n* = 677), with univariate association

Characteristic	Overall ED visits *n* = 833	Recurrent visit for hyperglycemia *n* = 156 (18.7%)	No recurrent visit for hyperglycemia *n* = 677 (81.3%)	*p* value*
Male (%)	455 (54.6)	88 (56.4)	367 (54.2)	0.62
Mean age, years (SD)	48.8 (19.5)	45.8 (22.4)	49.5 (18.7)	0.03
Range	18–95	18–88	18–95	
<25 years (%)	126 (15.1)	48 (30.8)	78 (11.5)	<0.01
Vital signs				
Mean heart rate, bpm (SD)	96.4 (21.6)	93.0 (19.6)	97.2 (21.9)	0.07
Heart rate > 110 bpm (%)	188 (22.6)	19 (12.2)	169 (25.0)	<0.01
Systolic blood pressure <90 mm Hg (%)	14 (1.7)	3 (1.9)	11 (1.6)	N/A
Systolic blood pressure 90–150 mm Hg (%)	635 (76.2)	133 (85.3)	502 (74.2)	<0.01
Systolic blood pressure >150 mm Hg (%)	184 (22.1)	20 (12.8)	164 (24.2)	<0.01
Temperature >38.0 or <36.0 °C on arrival (%)	192 (23.0)	28 (17.9)	164 (24.2)	0.09
Mean blood glucose mmol/L (SD)	27.6 (12.6)	29.0 (11.9)	27.3 (12.8)	0.14
Range	3.1–92	4.9–74.5	3.1–92	
>20 mmol/L (%)	557 (66.9)	119 (76.3)	438 (64.7)	<0.01
Arrival by EMS (%)	383 (46.0)	93 (59.6)	290 (42.8)	<0.01
CTAS 1 or 2 (%)	478 (57.4)	86 (55.1)	392 (57.9)	0.53
From nursing home or long-term care facility (%)	56 (6.7)	18 (11.5)	38 (5.6)	0.008
Previously known history of DM (%)	721 (86.6)	146 (93.6)	575 (84.9)	0.004
DM1	325 (39.0)	82 (52.6)	243 (35.9)	<0.01
DM2	396 (47.6)	64 (41.0)	332 (49.0)	0.07
New DM diagnosis in ED	112 (13.4)	10 (6.4)	102 (15.1)	0.004
Diabetic medications (%)				
Oral hypoglycemic	329 (39.5)	73 (46.8)	256 (37.8)	0.04
Subcutaneous insulin	481 (57.7)	115 (73.7)	366 (54.1)	<0.01
Insulin pump	47 (5.6)	10 (6.4)	37 (5.5)	0.65
Physicians (%)				
Family physician	711 (85.4)	147 (94.2)	564 (83.3)	<0.01
Internist or endocrinologist	354 (42.5)	87 (55.8)	267 (39.4)	<0.01
Past medical history (%)				
Hypertension	383 (46.0)	68 (43.6)	315 (46.5)	0.51
Hyperlipidemia	315 (37.8)	58 (37.2)	257 (38.0)	0.86
Coronary artery disease	138 (16.6)	28 (17.9)	110 (16.2)	0.60
Chronic renal failure	114 (13.7)	15 (9.6)	99 (14.6)	0.10
Stroke/transient ischemic attack	60 (7.2)	13 (8.3)	47 (6.9)	0.54
Psychiatric illness	291 (34.9)	81 (51.9)	210 (31.0)	<0.01
Intravenous drug abuse	34 (4.1)	4 (2.6)	30 (4.4)	0.29
Alcohol abuse	55 (6.6)	7 (4.5)	48 (7.1)	0.24

*SD* standard deviation, *bpm* beats per minute, *EMS* emergency medical services, *CTAS* Canadian Triage and Acuity Scale, *DM* diabetes mellitus.

*p value compares characteristic for those with vs. without a recurrent visit for hyperglycemia within 30 days

**Table 2:** Chief complaints of all 833 emergency department visits for hyperglycemia

Chief complaint	*n* = 833 (%)
High blood sugar	403 (48.4)
Dizzy, weak, and/or unwell	140 (16.8)
Nausea and/or vomiting	105 (12.6)
Decreased level of consciousness	46 (5.5)
Abdominal pain	31 (3.7)
Chest pain or palpitations	21 (2.5)
Shortness of breath	21 (2.5)
Polyuria and/or polydipsia	15 (1.8)
Other (infection, limb paresthesia, falls)	51 (6.1)

Table 3 lists the most likely precipitating causes of hyperglycemia among all 833 hyperglycemia visits. The most common causes were medication or insulin non-compliance (35.8%), ongoing poor control or under-dosing of medication or insulin (28.9%), and infection from various sources (21.7%). Of note, the emergency physician made a new diagnosis of diabetes mellitus in 12.1% of patients who were presenting to the ED with hyperglycemia, and alcohol-related causes were the precipitant for the hyperglycemic visit in 4.2% of all cases.

**Table 3:** Likely precipitant of hyperglycemia for all 833 emergency department visits

Precipitant*	*n* = 833 (%)
Medication or insulin non-compliance	298 (35.8)
Medication or insulin under-dosing/poor control	241 (28.9)
Infection	181 (21.7)
Respiratory	53 (6.4)
Urinary	46 (5.5)
Gastrointestinal	44 (5.3)
Other	38 (4.6)
New diagnosis of DM	101 (12.1)
Alcohol-related	35 (4.2)
Insulin pump problem	20 (2.4)
Acute coronary syndrome/cardiac ischemia	14 (1.7)
Other (corticosteroid related and pancreatic pathology)	46 (5.5)
Unknown	45 (5.4)

*DM* diabetes mellitus

*May have multiple precipitants of hyperglycemia

The final diagnoses, consultations, dispositions and 30-day outcomes of all 833 ED hyperglycemic patient visits are outlined in Table 4. The final discharge diagnosis was hyperglycemia or diabetes in 463 (55.6%), diabetic ketoacidosis in 288 (34.6%), and hyperosmolar hyperglycemic state in 79 (9.5%) of visitors. The vast majority of patients who had ED consultation for admission were referred to the internal medicine service. Physicians discharged 414 (49.7%) patients home from the ED, but 407 (48.9%) were admitted to hospital, with 389 of these admitted to the ward and only 18 to the ICU. One patient died in the ED, and a total of six died in hospital. Within 30 days of the index hyperglycemia visit, 156 (18.7%) had an unplanned return visit to the ED for hyperglycemia, 73 (8.8%) required hospital admission, and only two of these (0.2%) were admitted to the ICU. Of the 156 return hyperglycemia ED visits within 30 days, six also had urinary tract infections, five had cardiac ischemia, four had pneumonia, four had chronic abdominal pain, two had undifferentiated sepsis, and 18 had various “other” alternate diagnoses (including substance abuse, anxiety, dehydration, leg abscess, etc.). The remaining 117 patients did not have an alternate diagnosis reported and returned to the ED for hyperglycemia alone.

**Table 4:** Final diagnoses, consultations, disposition, and outcomes for all 833 emergency department hyperglycemia visits

Outcome	*n* = 833 (%)
Final hyperglycemic diagnosis	
Hyperglycemia or DM	463 (55.6)
Diabetic ketoacidosis	288 (34.6)
Hyperosmolar hyperglycemic state	79 (9.5)
Final physician diagnosis missing	3 (0.4)
Consultations in ED	
Internal medicine	378 (45.4)
Intensive care unit	35 (4.2)
Endocrinology	28 (3.4)
Other (family medicine, nephrology, cardiology, oncology)	31 (3.7)
Disposition from ED	
Discharged home	414 (49.7)
Admitted	407 (48.9)
To ward	389 (46.7)
To intensive care unit	18 (2.2)
Left against medical advice	11 (1.3)
Death in ED	1 (0.1)
Death in hospital	6 (0.7)
Return visits to ED for hyperglycemia	
Within 72 h	30 (3.6)
Within 7 days	48 (5.8)
Within 14 days	71 (8.5)
30-day outcomes	
Return visit to ED for hyperglycemia	156 (18.7)
Hospital admission for hyperglycemia	73 (8.8)
ICU admission for hyperglycemia	2 (0.2)

*DM* diabetes mellitus, *ED* emergency department, *ICU* intensive care unit

Univariate analysis of all potential risk factors were completed and clinically relevant variables with a *p* value of 0.10 or less in the univariate analysis were considered for the multivariate GEE regression model (Table 1). Variables with a high number of missing values were also excluded from the model. After adjusting for admission status, five risk factors were identified as predictors of the primary outcome (Table 5). Factors independently associated with a recurrent hyperglycemia visit within 30 days included a previous hyperglycemia visit in the past month (odds ratio [OR] 3.5, 95% confidence interval [CI] 2.1–5.8), age <25 years (OR 2.6, 95% CI 1.5–4.7), initial glucose >20 mmol/L on fingerstick or laboratory testing (OR 2.2, 95% CI 1.3–3.7), having a family physician (OR 2.2, 95% CI 1.0–4.6), and being on insulin (OR 1.9, 95% CI 1.1–3.1). Interestingly, having a systolic blood pressure between 90–150 mmHg (OR 0.53, 95% CI 0.30–0.93) and heart rate >110 bpm (OR 0.41, 95% CI 0.23–0.72) were factors independently associated with not having a recurrent hyperglycemia visit. The adjusted analysis did not show that those with type 1 diabetes were at higher risk for an unplanned ED visit compared to those with type 2 diabetes.

**Table 5:** Variables independently associated with 156 unplanned recurrent ED visits for hyperglycemia within 30 days as determined by multivariable logistic regression model and generalized estimating equations

Risk Factor	Beta Co-efficient	Standard Error	P value	Adjusted Odds Ratio	95% Confidence Interval
Previous hyperglycemia visit in past month	1.26	0.26	<0.01	3.5	2.1, 5.8
Age < 25 years	0.97	0.30	<0.01	2.6	1.5, 4.7
Glucose > 20 mmol/L	0.80	0.26	<0.01	2.2	1.3, 3.7
Have a family physician	0.77	0.39	0.04	2.2	1.0*, 4.6
On insulin	0.62	0.26	0.02	1.9	1.1, 3.1
Protective Factor					
Systolic blood pressure 90-150 mm Hg	0.63	0.29	0.03	0.53	0.30, 0.93
Heart rate > 110 bpm	0.90	0.29	<0.01	0.41	0.23, 0.72

Where *SE* standard error, *bpm* beats per minute

*Note*: No test for goodness-of-fit available under generalized estimating equation modeling

*The lower limit of the 95% confidence interval for having a family physician was 1.01

**Fig. 2:**
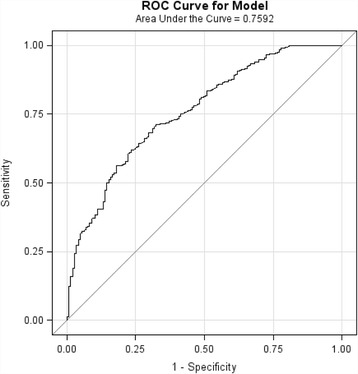
Receiver operating characteristic (ROC) curve for generalized estimating equation regression model. Area under the curve = 0.7592 (95% CI*: 0.7167, 0.8017) Note that 95% CI disregards clustering of data

The ROC curve for the GEE regression model is presented in Fig. 2. The area under the curve for the model was 0.7592 (95% CI 0.7167, 0.8017).

## Discussion

The results of this unique multicenter study may aid emergency physicians in making follow-up and disposition decisions for patients presenting with hyperglycemia, given the lack of evidence in this area at present. While the risk factors in this study require validation, they may represent variables that can be considered by clinicians when deciding which individuals may benefit the most from targeted medical or educational interventions to improve glycemic control.

Return ED visits for hyperglycemia represent negative outcomes for patients with diabetes and impact the healthcare system overall. A study by Sykes et al. demonstrated that the frequency of readmission to the critical care unit for recurrent diabetic ketoacidosis within 1 year was higher in those with older age, female gender, concurrent sepsis, anemia, and increased anion gap on admission [14]. However, this study was conducted in the ICU, making the conclusions difficult to generalize to ED patients who may not be as critically ill. Another study by Driver et al. determined that glucose levels at discharge and amount of glucose reduction were not associated with short-term adverse outcomes such as an unplanned return visit for hyperglycemia, diabetic ketoacidosis, hospitalization for any reason, and death within 7 days of initial presentation [21]. While the population in this study was ED patients, the authors only included hyperglycemic patients with type 2 diabetes and the results would not be generalizable to patients with type 1 disease.

In our study, patients who had already had a previous hyperglycemia visit within the past month were most at risk for a recurrent unplanned ED visit for hyperglycemia. It is unsurprising that individuals who may utilize the ED for management of a recurring, chronic condition such as diabetes are likely to do so on an ongoing basis. While there are studies on other disease processes such as asthma, cancer, psychiatric, or cardiac disease that describe past ED visits as being a risk factor for subsequent visits, to our knowledge, this is the first study that has highlighted this fact in a population presenting to the ED specifically for hyperglycemia [10–13, 22]. Additionally, it is to be expected that individuals presenting with a higher initial blood glucose (>20 mmol/L) are at increased risk of ED recidivism as these patients likely represent those who have ongoing poor glycemic control.

Although our study found that age <25 years and being on insulin were independently associated with our primary outcome, the adjusted analysis did not show that those with type 1 diabetes in general were at higher risk for an unplanned ED visit compared to those who had type 2 diabetes. It is likely that younger patients with type 1 diabetes are generally less compliant or less experienced with their disease and thus having more frequent unplanned ED visits, while older, type 1 patients are managing better in the community. Furthermore, patients with type 2 diabetes who are insulin-dependent tend to be less well-controlled than type 2 patients who are being managed with oral hypoglycemics only, and may be more likely to return to the ED more often. This appears to be the case since use of insulin tends to be delayed in all areas of clinical practice and irreversible complications can already be present by the time insulin therapy is initiated [23].

Patients who had a family physician listed on their electronic record were found to be at higher risk for recurrent unplanned ED visits for hyperglycemia in our study. As this finding was unexpected, it was verified by reviewing the original patient records, confirming correct coding in the study-specific database, and ensuring statistical accuracy. The lower limit of the 95% confidence interval for this risk factor was 1.01, confirming that having a family physician is indeed independently associated with the primary outcome in our study. It is entirely possible that patients with poorer glycemic control and diabetic complications are more proactive about having a regular physician to follow up with, whereas those with milder disease or better control may not feel the need to be rostered with a family physician. Unfortunately, our study is inherently limited with its retrospective nature and we were thus unable to confirm actual accessibility of follow-up despite having a family physician listed on the medical record. A future prospective study examining a patient’s ability to access follow-up with a healthcare provider and transition their care from emergency to primary care would help to determine if improved access to a family physician would lead to reduced unnecessary ED return visits for hyperglycemia. Indeed, studies of other chronic disease entities such as congestive heart failure or chronic obstructive pulmonary disease have demonstrated that access to follow-up is associated with a decreased 30-day risk of ED visits and readmission to hospital [24, 25]. Finally, we were unable to determine patients’ perceived urgency of their condition, which previous research has suggested may be important for patients in deciding whether to present immediately to the ED or to wait to see a family physician in a clinic setting [26, 27].

In this study, we found two protective factors associated with recurrent visits to the ED for hyperglycemia within 30 days. Firstly, patients who were normotensive (sBP 90–150 mmHg) were less likely to have the primary outcome compared to those who were either hypotensive or hypertensive. Furthermore, those with a heart rate >110 beats per minute were found to be less likely to re-present to the ED for hyperglycemia. Even after performing a sensitivity analysis and adjusting for admission to hospital from the index hyperglycemia visit, tachycardia was still found to be protective (unadjusted OR 0.42, 95% CI 0.24–0.74 vs. adjusted OR 0.41, 95% CI 0.23–0.72). It is likely that individuals presenting with tachycardia represented a population with a co-morbid disease process other than hyperglycemia and were thus less likely to return to the ED for poor glycemic control within 30 days.

## Limitations

Although the present study had a large sample size and was conducted at two academic centers consisting of four tertiary care EDs in Ontario, Canada, the results may not be generalizable to community settings or EDs outside of this geographical location. Additionally, it is possible that patients may have sought care at community EDs surrounding our study sites. However, if this were true, the outcome rates reported in this study would actually be an underestimate of the true incidence in the population. As a result, the occurrence of recurrent ED visits may in fact be more frequent than our results would suggest.

Due to the retrospective nature of this health records review, this study is limited by the data that were recorded on patient charts. It is possible that some patients in our study period were missed if the treating physician’s final diagnoses did not include an ICD-10 code related to hyperglycemia, diabetic ketoacidosis, or hyperosmolar hyperglycemic state, particularly if they were perceived to have a more important diagnosis such as cardiac arrest, acute coronary syndrome, or stroke. However, we attempted to mitigate this limitation by reviewing both primary and secondary diagnoses on all eligible ED visits. Furthermore, as previously mentioned, although we determined if patients had a family physician, internist, or endocrinologist, we were unable to ascertain if patients were able to access these healthcare professionals in follow-up after their ED visits unless they were seen within the hospital’s outpatient clinics. It is possible that those who were successful in seeing their physicians for follow-up may not have had to return to the ED for a subsequent hyperglycemia visit. Finally, a final discharge diagnosis of “hyperglycemia” without diabetic ketoacidosis or hyperosmolar hyperglycemic state may not be clinically meaningful or patient-important, especially if patients had a long history of poor control and were chronically hyperglycemic.

## Conclusions

This unique exploratory ED-based study reports five risk factors and two protective factors independently associated with unplanned recurrent ED visits for hyperglycemia within 30 days in patients with diabetes. Although these variables do not imply causation—but rather correlation—with repeat hyperglycemia visits, they should be considered when making management, prognostic, and disposition decisions for diabetic patients who present with hyperglycemia. Future prospective research should focus on confirming these factors and assess for additional correlates such as patient accessibility to follow-up and medication adjustments and compliance. Ultimately, a decision tool to aid clinicians in identifying those at higher risk for medical morbidity may be developed, and specific individual and system-based interventions may be of benefit for those who are identified to be at higher risk.

## Additional file

Additional file 1: Risk Factors and Management Practices of Recurrent Emergency Department Visits for Hyperglycemia in Patients with Diabetes Mellitus CASE RECORD FORM. (DOCX 39.2 kb)

## Abbreviations

95% CI: 95% confidence interval
ED: Emergency department
GEE: Generalized estimating equations
ICD-10: International Statistical Classification of Diseases and Related Health Problems, 10th Revision
ICU: Intensive care unit
OR: Odds ratio
ROC: Receiver operating characteristic
