# Supplementary material for: Risk factors for recurrent emergency department visits for hyperglycemia in patients with diabetes mellitus
Source: Int J Emerg Med. 2017 Jul 12;10:23. doi: 10.1186/s12245-017-0150-y (PMC5507935; doi:10.1186/s12245-017-0150-y)
Supplement: Additional file 1: — Risk Factors and Management Practices of Recurrent Emergency Department Visits for Hyperglycemia in Patients with Diabetes Mellitus CASE RECORD FORM. (DOCX 39.2 kb) [file 12245_2017_150_MOESM1_ESM.docx]

**Risk Factors and Management Practices of Recurrent Emergency Department Visits for Hyperglycemia in Patients with Diabetes Mellitus**

**CASE RECORD FORM**

**Case #:** _________

**Verification:**  Research Assistant _________ Principal Investigator _________

Coordinator _________ Data Entry _________

**DEMOGRAPHICS:**

Site: LHSC – UH ☐ LHSC – VH ☐ TOH-Gen ☐ TOH-Civic ☐

Date of visit: (yy/mm/dd) ________/________/________

Date of birth: (yy/mm) ________/________

Sex: Male ☐ Female ☐

Postal Code: __________________________ No Fixed Address ☐ Nursing Home/Long-term care ☐

**EMERGENCY DEPARTMENT EVALUATION:**

Arrival Mode: EMS ☐ Self ☐

CTAS: (1-5) _________

Triage Time: (00:00-23:59) _________

Physician Assessment Time: (00:00-23:59) _________

Discharge Time: (00:00-23:59) _________

**INITIAL CLINICAL PRESENTATION:**

Main Presenting Symptom (1 only): All Symptoms (check any)

High Blood Sugar ☐ ☐

Decreased LOC ☐ ☐

Fever ☐ ☐

Nausea/vomiting ☐ ☐

Abdominal Pain ☐ ☐

Dizzy/weak/unwell ☐ ☐

Short of breath ☐ ☐

Chest Pain ☐ ☐

Polyuria/dipsia ☐ ☐

Other ________________ ☐ ☐

Temp: ________ sBP: ________ HR: ________ RR: ________ SaO2: ________ on O2 Yes ☐ No ☐

POC Blood glucose: Home ________ EMS ________ Initial ED _________

EMS interventions: Yes ☐ No ☐

If yes: IV fluid: Yes ☐ No ☐

Antiemetic: Yes ☐ No ☐

Analgesia: Yes ☐ No ☐

Other Medication: Yes ☐ No ☐ ____________________________

Oral Airway: Yes ☐ No ☐

BVM: Yes ☐ No ☐

Intubation: Yes ☐ No ☐

Other Intervention: Yes ☐ No ☐ ____________________________

**PAST MEDICAL HISTORY AND MEDICATIONS:**

Known History of Diabetes: Yes ☐ No ☐ Type: 1 ☐ 2 ☐

On insulin? Yes ☐ No ☐ If yes, on insulin pump? Yes ☐ No ☐

Insulin taken today? Yes ☐ No ☐ Unknown☐

On oral hypoglycemic? Yes ☐ No ☐

If yes: Metformin? Yes ☐ No ☐

Glyburide (Diabeta) Yes ☐ No ☐

Gliclazide (Diamicron) Yes ☐ No ☐

Rosiglitazone (Avandia) Yes ☐ No ☐

Pioglitazone (Actos) Yes ☐ No ☐

Sitagliptin (Januvia) Yes ☐ No ☐

Other: Yes ☐ No ☐

Comorbidities:

Hyperlipidemia Yes ☐ No ☐

Hypertension Yes ☐ No ☐

Coronary artery disease (CAD) Yes ☐ No ☐

Chronic renal failure (CRF) Yes ☐ No ☐

Peripheral vascular disease (PVD) Yes ☐ No ☐

Stroke/TIA Yes ☐ No ☐

Asthma/COPD Yes ☐ No ☐

Psychiatric illness Yes ☐ No ☐

IVDU Yes ☐ No ☐

Alcohol Abuse Yes ☐ No ☐

Pregnancy Yes ☐ No ☐

Cancer and type _______________ Yes ☐ No ☐

Other Medications:

Blood Pressure Yes ☐ No ☐

Cholesterol Yes ☐ No ☐

Cardiac Yes ☐ No ☐

Steroids Yes ☐ No ☐

Antibiotics Yes ☐ No ☐

Has Family Doctor Yes ☐ No ☐

Has Internal Medicine Yes ☐ No ☐

Has Endocrinologist Yes ☐ No ☐

DM Education Nurse Yes ☐ No ☐

**INVESTIGATIONS**:

Bloodwork:

CBC: Hgb __________ WBC __________

Electrolytes: Na __________ K __________ Cl __________ CO2 __________

Anion Gap __________ Glucose __________

BUN __________ Creatinine __________

Blood Gas: Arterial ☐ Venous ☐

pH __________ pCO2 __________ pO2 __________ HCO3 __________

Serum ketones or beta-hydroxybutyrate __________

Lactate __________

Urinalysis: Ketones: Yes ☐ No ☐ Protein: Yes ☐ No ☐

Leuks: Yes ☐ No ☐ Glucose: Yes ☐ No ☐

Nitrites: Yes ☐ No ☐ Blood: Yes ☐ No ☐

ECG: Rhythm __________________ Ischemic Changes: Yes ☐ No ☐

Imaging:

Chest xray: Yes ☐ No ☐

If yes, Normal: ☐ Pneumonia: ☐ CHF/Pulmonary Edema: ☐

Other: Yes ☐ No ☐ ____________________

CT head: Yes ☐ No ☐

If yes, Normal: ☐ Cerebral edema: ☐ Ischemic stroke: ☐

Intracranial hemorrhage: ☐ Other: ☐ ____________________

**EMERGENCY DEPARTMENT MANAGEMENT:**

ED Hyperglycemia Interventions:

Oral hypoglycemic administered: Yes ☐ No ☐

Type/dose: ____________________________________

Insulin **bolus** administered: Yes ☐ No ☐

Method: IV ☐ SC ☐

Type/dose: ____________________________________

Insulin **infusion** administered: Yes ☐ No ☐

Type/dose: ____________________________________

Fluids administered: Yes ☐ No ☐

Amount: ____________________________________

Sodium Bicarbonate administered: Yes ☐ No ☐

Amount: ____________________________________

ED Supportive Care Interventions

Airway Intervention/Intubated: Yes ☐ No ☐

Inotropes: Yes ☐ No ☐ Type: __________________

Other: Yes ☐ No ☐ Type: __________________

Consultations in the ED:

Medicine: Yes ☐ No ☐

ICU: Yes ☐ No ☐

Endocrine: Yes ☐ No ☐

Other: Yes ☐ No ☐ _____________________

Final Hyperglycemic Diagnosis (1 only):

Hyperglycemia/DM ☐ DKA ☐ HHS ☐

Other: ☐ ­­­_____________________

Alternate Diagnoses (check any):

UTI ☐ Pneumonia ☐ Sepsis ☐

Cardiac/coronary artery disease ☐ Other ☐ _____________________

Disposition (1 only):

LAMA ☐ Discharge ☐ Admitted to ward ☐ Admitted to ICU ☐ Death in ED ☐

If admitted: Survived to hospital discharge ☐ Death in hospital ☐

Likely precipitant of hyperglycemia (check any)

Insulin related/control ☐

Non-compliance ☐

New diagnosis of DM ☐

Infection ☐

Respiratory ☐ Urinary ☐ GI ☐

Neurologic ☐ Skin/Soft tissue ☐ Genital/Gyne ☐

Cardiac Ischemia ☐

Other ☐ _______________________

Discharge instructions: Yes ☐ No ☐

Follow up ☐

Family Physician ☐ Internal Medicine ☐

Endocrinology ☐ DM Education RN ☐ Other ☐ ________________

Medication change ☐

Insulin ☐ Oral hypoglycemic ☐

Prescription ☐

Insulin ☐ Oral hypoglycemic ☐ Antibiotics ☐

Other ☐ _______________________________________________________________________________

**30-DAY OUTCOMES (ALL PATIENTS):**

Return visit to ED for hyperglycemia within 30 days: Yes ☐ No ☐

Hospital admission for hyperglycemia within 30 days: Yes ☐ No ☐

ICU admission for hyperglycemia within 30 days: Yes ☐ No ☐

**PREVIOUS “SENTINEL” ED VISIT FOR ANY REASON**

Within past 14 days: Yes ☐ No ☐

Date of visit: (yy/mm/dd): ________/________/________

POC Blood glucose Documented: Yes ☐ No ☐ If yes, level: _________

Final Diagnosis:

Hyperglycemia ☐ Sepsis ☐

DKA ☐ Pneumonia ☐

HHS ☐ UTI ☐

Cardiac/coronary artery disease ☐

Other ☐ ______________

Disposition:

LAMA ☐ Discharge ☐ Admitted to ward ☐ Admitted to ICU ☐

Discharge instructions: Yes ☐ No ☐

Follow up ☐

Family Physician ☐ Internal Medicine ☐

Endocrinology ☐ DM Education RN ☐ Other ☐ ________________

Medication change ☐

Insulin ☐ Oral hypoglycemic ☐

Prescription ☐

Insulin ☐ Oral hypoglycemic ☐ Antibiotics ☐

Other ☐ _______________________________________________________________________________

**PREVIOUS VISITS FOR HYPERGLYCEMIA**

In past 1 month : Yes ☐ No ☐

If yes: Disposition: ED visit only ☐ Admission ☐ ICU Admission ☐

Diagnosis: Hyperglycemia ☐ Sepsis ☐ DKA ☐ Pneumonia ☐ HHS ☐ UTI ☐ Cardiac/coronary artery disease ☐

Other ☐ ______________

In past 6 months Yes ☐ No ☐

If yes: Disposition: ED visit only ☐ Admission ☐ ICU Admission ☐

Diagnosis: Hyperglycemia ☐ Sepsis ☐

DKA ☐ Pneumonia ☐

HHS ☐ UTI ☐

Cardiac/coronary artery disease ☐

Other ☐ ______________

In past 12 months Yes ☐ No ☐

If yes: Disposition: ED visit only ☐ Admission ☐ ICU Admission ☐

Diagnosis: Hyperglycemia ☐ Sepsis ☐

DKA ☐ Pneumonia ☐

HHS ☐ UTI ☐

Cardiac/coronary artery disease ☐

Other ☐ ______________
